# Handover Scheme in LEO Satellite Networks Based on QoE for Streaming Media Services

**DOI:** 10.3390/s25072165

**Published:** 2025-03-28

**Authors:** Huazhi Feng, Lidong Zhu

**Affiliations:** National Key Laboratory of Wireless Communications, University of Electronic Science and Technology of China, Chengdu 611731, China; fenghz@std.uestc.edu.cn

**Keywords:** streaming media, QoE, LEO satellite, handover

## Abstract

The development of satellite communications has received considerable attention in recent years. Early satellite communications were dominated by voice and low-speed data services, but now they must support high-speed multimedia services. Low Earth Orbit (LEO) satellites, because of their lower altitude orbits, have much smaller transmission loss and delay than Geostationary Earth Orbit (GEO) satellites, and they are an important part of the future realization of high-bandwidth and low-latency multimedia services. Among them, the on-demand streaming service has a large number of users in terrestrial communication and is also an important service component that will be in satellite communication environments in the future. However, LEO satellites face many challenges in handover and accessing due to their fast moving speed. Although many handover and access schemes for LEO satellites have been proposed and evaluated in existing studies, most of them stay at the level of quality of service (QoS), and few of them have been studied at the level of quality of experience (QoE). These studies also rarely consider the performance of multimedia services, including streaming services, in satellite communication environments, and there is no relevant simulation system to evaluate and examine them. Therefore, this paper builds a simulation system for streaming services in LEO satellite communication environments in order to simulate the initial buffering, rebuffering, and idle state of the users during service. Then, access and handover schemes for the QoE level of streaming service are proposed. Finally, our proposed scheme is evaluated based on this simulation system. From the simulation results, the simulation system proposed in this paper can successfully realize the various functions of users in on-demand streaming services and record the initial buffering and rebuffering events of users. And the streaming QoE-based access and handover scheme proposed in this paper can perform well in satellites, which operate within a resource-constrained environment.

## 1. Introduction

With the development and popularization of 5G technology, more and more users are enjoying higher quality communication services. Compared with the characteristics of 5G, such as ultra-low latency, high reliability, ultra-high speed and massive terminal access, the future 6G must solve another problem—coverage [1]. However, providing full coverage via fixed terrestrial cellular infrastructure is prohibitively costly in sparsely populated regions. To overcome this limitation, integrating non-terrestrial networks (e.g., satellites) has emerged as a key strategy for 6G evolution [2]. For instance, large-scale LEO constellations (e.g., OneWeb and Starlink) have been deployed and are already operational. In addition, Huawei’s Tiantong Satellite (geosynchronous orbit satellite) mobile phone direct call service [3], in cooperation with China Telecom Group and other enterprises, has been put into commercial use, making it possible to use 2G services in some areas without cellular network coverage. Beyond basic calls and SMS, future satellite communications aim to deliver multimedia services and ubiquitous Internet access [4]. Multimedia services will have higher requirements on QoS metrics such as transmission rate and delay [5], and different multimedia services will have different requirements on QoS metrics due to different QoE requirements. Unlike QoS, which measures the performance of communications or the network themselves, QoE captures users’ subjective perception of service quality, encompassing factors such as effectiveness, availability, and application-specific comfort levels.

As one of the important components of the multimedia service, an on-demand streaming media service has special requirements for QoS metrics such as bandwidth to meet users’ QoE needs due to its download-while-playback and buffering mechanisms. However, QoE-centric studies of on-demand streaming media in satellite environments remain scarce. In addition, satellites are resource-constrained platforms with limited power and bandwidth, and the orbital velocity of LEO satellites is very fast, which makes streaming media transmission very different from fixed base stations on the ground. Therefore, it is of great significance to establish a simulation system for streaming media transmission under the satellite platform and to improve the QoE of users by the rational allocation of satellite resources according to the current satellite parameters.

Existing simulation studies primarily focus on QoS-level evaluations. For example, both [6,7] work on performance evaluation of the 3GPP-compliant 5G conditional handover (CHO) in LEO-based non-terrestrial networks. Ref. [6] verified some QoS performance of CHO as specified in 3GPP Rel-16 in non-terrestrial network (NTN) systems, such as Signal-to-Interference-and-Noise Ratio (SINR), the number of handover failures, the dwell time, and the ping-pong effect. In contrast, ref. [7] compares the traditional measurement-based handover triggering mechanism with other alternatives (e.g., based on distance, elevation angle, etc.) according to the 3GPP model for system-level simulation and performance comparison. The performance evaluation criteria are again limited to the QoS level. As for the research on schemes for LEO satellite handover, ref. [8] proposed an algorithm for channel-preserving load-aware handover to enhance the QoS and overall system satisfaction. Based on the three-attribute optimal handover problem, the optimal handover scheme is searched by combining the idea of reserving channels for important users. Ref. [9] leveraged the relatively low velocity of high-speed trains (compared to LEO satellites) to design an integrated terrestrial–satellite network, mitigating channel fluctuation and handover frequency in traditional terrestrial systems. Ref. [10] observed that users exhibit time-varying service preferences and proposed a user dynamic preference-based LEO satellite handover strategy for users on airplanes. A dynamic preference model is constructed based on different time periods, and attribute decision making is used to select the best handover satellite. Ref. [11] addresses the frequent handover challenges in Low Earth Orbit (LEO) satellite networks by leveraging Long Short-Term Memory (LSTM) to predict satellite motion patterns and implement dynamic handover decision making. Ref. [12] proposes the second satellite selection algorithm that predicts the service time, link quality, and expected handover time based on the visible satellite information and selects the optimal selection combination considering the second satellite. This algorithm improve user’s QoS and reliability when compared to other benchmark schemes. However, QoS does not fully reflect the user’s QoE. For instance, while higher transmission rates (a QoS metric) may enhance throughput, they do not improve the QoE if no rebuffering occurs during playback. In addition, among the QoS indicators of satellites, handover failure seriously affects the quality of QoS. For buffered streaming media services, transient handover failures may not degrade QoE provided the buffer remains non-empty. The factors affecting QoE for streaming services are discussed in Section 3.

For the QoE guarantee of streaming media services on the ground, considering the extremely complex and variable communication environment on the ground, there are two main types of schemes, which are optimized for bandwidth and buffer to optimize the QoE. Ref. [13] proposed to improve the QoE of video streaming through bandwidth allocation that is DASH-aware. Other work used bandwidth prediction [14] or predicted cellular network throughput for improved bitrate selection [15,16] to maximize over a QoE metric. Meanwhile, other studies consider optimization for buffers. Refs. [17,18] used buffer-based algorithms that select the optimal download bitrate based on buffer occupancy. In contrast, MPC [19], Pensieve [20], Oboe [21], etc., select bitrates as a function of buffer length and network throughput. However, in the LEO communication environment, the communication channel is more predictable than the terrestrial channel, especially in environments where satellite communication is needed, such as deserts, wilderness, oceans, etc. Therefore, the elevation angle change rule can be fully utilized to predict the channel to a certain extent so as to allocate reasonable communication resources under the circumstance of guaranteeing the QoE of users.

In this paper, we consider the scheme design of access and handover of the user’s streaming media service when it is transmitted in the system based on LEO satellite communication from the level of QoE. A simulation system is designed for a user’s streaming media service provider to transmit streaming media packets using LEO satellites, and the flow from transmission to the playback of video or audio services is realized. The system consists of four parts representing the four states of the user, i.e., idle, playback, initial buffering, and rebuffering, in order to simulate various environments of the user during the use of the streaming service. We propose QoE-driven access and handover algorithms tailored to three user states (playback, initial buffering, and rebuffering) by leveraging streaming buffering mechanisms, thereby ensuring the QoE under resource-constrained satellite networks. Unlike QoS-based methods, our scheme adapts to streaming media states. Specifically, it predicts the channel capacity to estimate changes in buffer occupancy at user terminals, enabling optimal resource allocation to prevent buffer exhaustion while reserving bandwidth for critical handover events during network congestion. Finally, we evaluated the proposed scheme’s performance using the simulation system and quantified QoE via Mean Opinion Score (MOS) comparisons against traditional handover methods. The main contributions of this paper are as follows:

- A simulation system for streaming media services under the LEO satellite communication environment is built to simulate the initial buffering, rebuffering, playback, and idle state of the user during the service and can reflect the initial buffering duration, rebuffering duration, number of rebuffering, etc., which affect the user’s QoE during the service.
- QoE-driven resource allocation algorithms for access and handover in LEO satellite systems, leveraging channel capacity prediction to estimate buffer occupancy changes and allocate resources, ensuring uninterrupted streaming while preserving bandwidth for critical handovers under network congestion.
- For cases where there are too many users leading to a shortage of LEO communication resources, we design the power occupation handover algorithm to allow other users to consume data in their own buffers instead of forcing the user whose playback is about to be stopped to continue to consume data in his or her own buffer.

## 2. System Model

**Figure 1:**
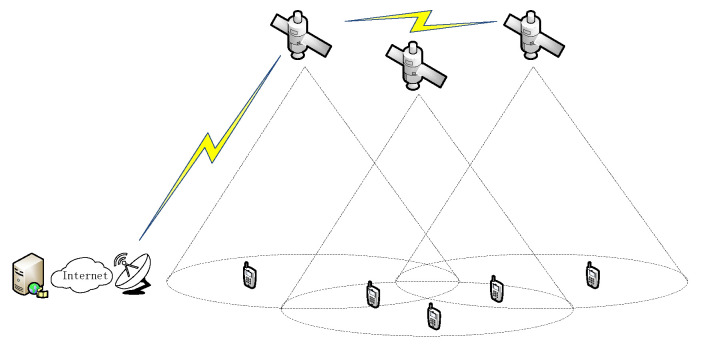
Multi-user LEO satellite communication system.

Considering that the flight speed of the LEO satellite is much higher than the moving speed of the user terminal on the ground, it can be assumed that the user terminal is stationary with respect to the Earth’s surface. As shown in Figure 1, the communication system consists of LEO satellites, user terminals, gateway station and terrestrial networks. The terminals are distributed in open environments on the surface (deserts, oceans, no man’s land, etc.), and their areas can be covered by multiple LEO satellites. Multiple users apply for multimedia services from the streaming server via terminals and satellite links. After a series of processes to establish the communication service, the server sends the data packets to the gateway station through the Intel satellite network, and then the gateway station sends the data packets to the LEO satellites linked by the users through the satellite–earth and inter-satellite links, and finally it sends them to the user’s terminal via the satellite–earth link. When the user uses the satellite communication service, the terminal decides whether to handover and to which candidate satellite by periodically measuring the received signal strength indication of the satellites within the coverage area and analyzing their satellite status parameters (elevation angle, remaining visible time, satellite load, etc.) as well as combining with the user’s own business status.

### 2.1. Downlink Budget Model

When user terminals can only use satellite multimedia services, they are usually in areas without terrestrial base stations, such as deserts, wildernesses, and oceans, so the free space propagation loss model is considered. Referring to the International Telecommunication Union (ITU) recommendation, when the frequency is within 100 GHz, the signal will also be affected by oxygen and water vapor attenuation, cloud attenuation, rainfall attenuation and tropospheric scintillation in the atmosphere [22]. These attenuations are related to the elevation angle θ. Therefore, the loss factors in this paper are summarized as follows:

To throw out other effects such as coding, we consider Shannon’s theorem to measure the effect of signal loss on the communication rate. The transmission losses listed in Table 1 are included in Shannon’s law, as shown in Equation (1):

(1) $$C=B\log_{2}\left(1+\frac{P_{T}G_{T}G_{R}}{L_{f}A_{g}A_{c}A_{r}A_{s}N}\right),$$

where $P_{T}$ is the satellite transmit power, $G_{T}$ is the transmit antenna gain, $G_{R}$ is the receive antenna gain, *N* is the noise power, *B* is the signal bandwidth, and *C* is the channel capacity. $A_{g}$ is the attenuation due to atmospheric gases, and for signals with frequencies below 100 GHz, it is mainly affected by oxygen and water vapor; thus, $A_{g}=A_{o}+A_{w}$. Note that the attenuation coefficients, gain coefficients, etc. are not in dB form here. When $G_{T}$, $G_{R}$, *B*, frequency *f*, noise *N*, and other factors in the atmosphere are constant, and the channel capacity is a function that varies with the elevation angle θ and the satellite transmit power $P_{T}$, that is

(2) $$C=f\left(P_{T},t\right)=B\log_{2}\left(1+\frac{G_{tot}}{N}\cdot \frac{P_{T}}{L_{t}\left(t\right)}\right),$$

where $G_{tot}$ is the total gain that includes the transmit antenna gain $G_{T}$ and receive antenna gain $G_{R}$, and *N* is the noise power. They are constants independent of θ and $P_{T}$. $L_{t}\left(t\right)$, which includes free space propagation loss and other attenuation such as atmospheric oxygen, water vapor, clouds, etc., is the value of the loss affected by the elevation angle θ(t). And the prediction of the elevation angle θ(t) as a function of the satellite service time *t* can be referred to the literature [23]. Therefore, the link loss $L_{t}\left(t\right)$ here is a function of the satellite service time *t*. If other effects such as coding are subsequently added for consideration, the coding efficiency can be expressed by multiplying a factor on the channel capacity *C*.

### 2.2. Streaming Media Transport Model

**Figure 2:**
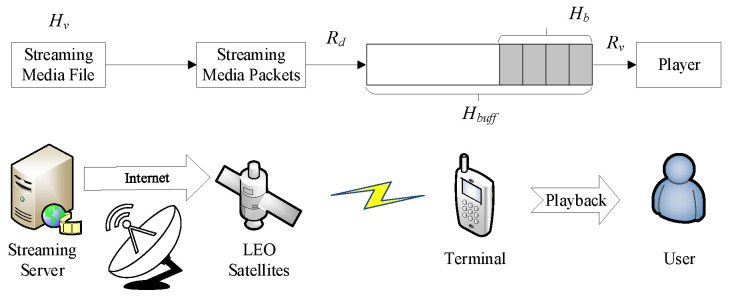
Streaming media transmission in satellite communication.

Streaming media technology refers to a series of media data compression in order to achieve progressive download (PD) transmission to the user terminal and realize the real-time transmission of audio and video on the network of a technology. Streaming media services can be further classified as on-demand streaming and live streaming. Common web video/music playback all belong to on-demand streaming media services, while web live or client live is live streaming media services. In this paper, we consider the handover of on-demand streaming services in satellite communications, as shown in Figure 2.

In Figure 2, $H_{v}$ is the size of the streaming media file, whose unit is bit; $R_{d}$ is the transmission rate from the satellite to the terminal and its unit is bps; $R_{v}$ is the bit rate of the media file, which is related to the resolution of the video or the sound quality of the audio as well as the compression scheme, and the unit is bps; $H_{b}$ is the playback threshold of the media file, whose unit is the same as $H_{v}$; $H_{b}$ is the amount of data occupied by the specified streaming media duration $T_{b}$ (in seconds); $H_{buff}$ is the buffer capacity, and its unit is the same as $H_{v}$. Streaming media files, such as video or audio, are compressed by streaming media-specific encoding and then transmitted from the server in the form of streaming media packets through the NTN to satellites and finally directly to user terminals through the satellite-terrestrial network. When a streaming media file is transmitted to a terminal, it is first stored in a preloaded area (called buffering) for a period of time. When the duration of the video in its buffer exceeds the playback threshold $H_{b}$, the file begins to play. During media playback, streaming data packets are sequentially extracted from the buffer, decoded, and rendered for user viewing. The buffer depletion rate precisely matches the media bitrate, while concurrent satellite downlink transmission continuously replenishes the buffer, enabling real-time streaming functionality through simultaneous download and playback. Because the streaming data generated by compression coding are in blocks, the buffer must have at least one complete block of data to play. If the average transmission speed is less than the bit rate of the video, the buffer will be depleted after a period of time, and the application will pause the media playback and rebuffer it until the amount of data in the buffer reaches the playback threshold again. If the remaining playback time of a video or audio file is less than the playback threshold, then the remaining video or audio content will be loaded before reaching the threshold and played directly.

## 3. Simulation System Design for Handover and Access of Streaming

**Figure 3:**
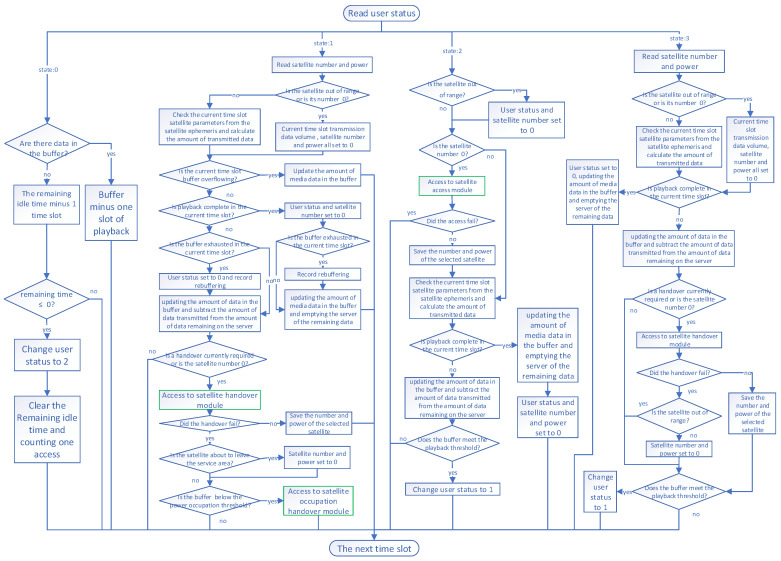
Streaming service simulation algorithm structure.

In this section, we design the access and handover algorithm for the QoE indicators of on-demand streaming media service in the subscriber communication service, and we simulate the entire service process to check the QoE performance of the proposed algorithm. Figure 3 is the algorithm structure diagram of the simulation we have built, which consists of four parts representing the four states of the user, which are idle (represented by state 0), playback (represented by state 1), initial buffering (represented by state 2), and rebuffering (represented by state 3). The green box shows the two algorithm modules for accessing and handover designed in this paper. The simulation process is divided into multiple time slots, and each time slot performs the simulation process once for each user.

- The user in an idle state is in the gap between streaming media playback, and the user may pause the playback service for a period of time because he/she is looking for a new video of interest before ordering a new streaming media. When the idle time is over, the user is handed over to the initial buffering state.
- The user is in the playback state when the service is being used. The satellite downlink transmits streaming packets from the satellite to the buffer at the user’s terminal, while the user plays the streaming files in the buffer in chronological order. This state will determine whether the user’s service is stalled, whether the buffer is overflowing, and whether the playback is complete and other conditions so as to change the user’s state. When the streaming data in the buffer of the user terminal are below the handover threshold $H_{Thre}$ and at the same time, the satellite elevation angle is decreasing, the system starts trying to handover. If none of the candidate handover satellites satisfy the condition, then the current time slot is abandoned for handover, and the handover will wait for the next time slot. When the satellite handover fails several times, and the buffer is below the power occupation threshold $H_{oc}$, it begins to enter the occupancy handover. The occupancy handover takes up a portion of the other user’s power, allowing the current user to meet the minimum power requirement.
- The user in the initial buffering state is in the initial buffering phase after having just ordered a streaming service. The user will not play the video at this stage. First, the system will determine whether a suitable satellite has been selected. If not, it will enter the satellite access module. Then, the system sends the streaming media packet to the buffer. When the data in the buffer exceed the playback threshold, it transfers to the playback state.
- The user in the rebuffering buffer state is switched to this state after the buffer is exhausted in the playback state. In this state, the user stops playback and starts buffering until the buffer fills up to the playback threshold. Finally, it will detect if a handover is needed, usually after repeated failed handovers lead to buffer exhaustion, which then causes playback to stop. At this point, the system will attempt to handover at each time slot until playback resumes.

In this paper, we design the QoE-based handover and access scheme for streaming services, so we must first consider how the QoE of streaming services is evaluated. For the evaluation of QoE in this type of service, there are existing papers using their own evaluation methods [19]. Given that a QoE assessment is highly subjective, this paper therefore refers to the ITU-T P.1201 assessment model [24], which is capable of estimating the quality of Progressive Download (PD) streaming applications; i.e., it is used for the quality prediction of non-adaptive TCP-based streams. The model predicts the Mean Opinion Score (MOS) [25] on a 5-point Absolute Category Rating (ACR) scale as the global multimedia MOS. This scheme to measure the QoE of streaming media is often used in multimedia research [26].

The model evaluates the performance and quality of real-time networks (including codecs) for audio, video, and audiovisual, including the impact of coding bitrate and transmission issues that lead to long initial buffering and stalling events. Considering that metrics such as bitrate are also related to the choice of multimedia application providers and the users themselves, this paper only considers the model’s impact on the MOS score of initial buffering and stalling events in the middle of playback to react to the user’s QoE.

Quality impacts DegT0 from initial buffering

(3) $$DegT0=\left\{\begin{array}{cc} \max(\min\left(d_{1}\times \mathrm{lg}\left(T_{0}+d_{2}\right),4\right),0) & T_{0}>1-d_{2}\\ 0 & else \end{array}\right.,$$

where $T_{0}$ is the initial loading time of the video in seconds. $d_{1}$ and $d_{2}$ are fixed coefficients whose values are referenced in Table 2. The impact of stalled buffering events in playback on quality DegStall is

(4) $$DegStall=max\left(min\left(s_{4}+s_{1}\times exp\left(\left(s_{2}\times L+s_{3}\right)\times N\right),4\right),0\right),$$

where *L* is the average rebuffering time in seconds (excluding the initial loading time), and *N* is the number of stalls occurring (excluding the initial buffer). The values of the coefficients of Equations (3) and (4) are given in Table 2. These coefficients are fitted and validated based on the ITU database-set; refer to [24] for details.

The final evaluation score PBufInd (presented on a scale of 1 to 5) for video buffering wait is calculated as follows:

(5) $$PBufInd=5-max\left(min\left(\left(DegStall+DegT0\right),4\right),0\right)$$

As can be seen from the evaluation system above, the reception rate of the user terminal is directly associated with the streaming QoE. The magnitude of the reception rate will directly affect the initial loading time $T_{0}$ of the video or audio and the average rebuffering time L during playback, thus reducing the user’s video playback experience. At the same time, from the exponent in Equation (4), it can be inferred that if the quality of the user streaming service is to be guaranteed, the average user download rate should be as high as possible above the bit rate of streaming media to avoid data exhaustion in the buffer; otherwise, the user will suffer playback rebuffering, which will lead to a significant decrease in QoE [27]. The strategy of many streaming media content platforms is also to try their best to avoid mid-play stagnation [28], and these platforms are designed to have redundancy of up to 30%~40% of the transmission rate; in other words, when the platform detects that the current reception rate is not higher than 130%~140% of the current video bitrate, it will immediately reduce the resolution of the video itself to lower the video’s bitrate. In this paper, we will design the access and words; when the platform detects that the current reception rate is not higher than 130%~140% of the current video bitrate, it will immediately reduce the resolution of the video itself to lower the video’s bitrate. In this paper, we will design the access and handover algorithms according to the characteristics of these streaming media services, and we will also provide some redundancy in the transmission rate, considering that satellite environments are more stable than terrestrial, so the access and handover scheme in this paper is set at 10% redundancy in channel capacity.

### 3.1. Handover Scheme Design Based on Streaming Services

To leave redundancy for handover failures, we set the handover to start when the data in the buffer are below the handover threshold $H_{Thre}$. When the handover is about to take place, it is taken into account that the satellite environment is different from the terrestrial environment. The channel on the ground is almost unpredictable, so the strategy on the ground is to adopt the strategy of obtaining the maximum transmission rate possible and then filling the buffer [28]. In contrast, in satellite communication environments, satellite resources are limited while the channel is relatively stable and predictable. If the optimization is based on QoS indicators (such as bandwidth, delay, etc.) and does not take into account the characteristics of streaming media services, then an excessive improvement of QoS may lead to communication performance overflow and a waste of satellite resources. In addition, multi-user contention for maximum bandwidth resources may also lead to a “downward spiral effect” [28]. Therefore, our access scheme is based on the QoE of streaming media.

From Section 2.1, the channel capacity of the satellite to be linked by the user can be calculated for the entire service cycle with known power and satellite status. And from the analysis in the previous section, it can be seen that to ensure the user’s QoE in streaming media playback, it is necessary to ensure that there is no rebuffering during the service. Therefore, the channel capacity during the user’s access to the satellite needs to meet the following two conditions:

- The average channel capacity from the time of access to the satellite until the satellite has flown to its highest elevation angle is not less than the bit rate required for the user’s streaming service.
- The buffer shall not be exhausted from the time of access to the satellite until the satellite reaches its highest elevation angle.

Consider the first condition first. For the handover user, the elevation angle and trajectory of the currently accessible satellite are fixed, so the only parameters that the user can choose are the access power and which satellite. Suppose that the set $S_{i}=\left\{S_{i,1},S_{i,2},...,S_{i,j},...\right\}$ is the set of all satellites within the access range of the user $u_{i}\in \left\{u_{1},u_{2},...,u_{i},...\right\}$. In our paper, it is assumed that the total downlink power resource of each satellite in the system is $P_{tot}$. Considering that the transmission rate of user services is guaranteed as much as possible, the range of selectable satellites is limited to the moments when the elevation angle is above $10^{{}^{\circ}}$ and is in elevation rise. For a satellite $S_{i,j}$, its service time is $\left[\theta ,t_{max}\right]$, where $t_{max}$ is the length of time from when the satellite enters the service range to when the elevation angle is at its maximum [23]. A handover is initiated for user $u_{i}$ at the time $t_{acc}$ when the elevation angle of the satellite $S_{i}$ relative to the user is $\theta_{acc}$ ($\theta_{acc}>10^{{}^{\circ}}$). Assuming that the power of the user’s access to the satellite $S_{i,j}$ is $P_{i,j}$, the channel capacity variation brought into Equation (2) is

(6) $$C\left(t,P_{i,j}\right)=B\log_{2}\left(1+\frac{G_{tot}}{N}\cdot \frac{P_{i,j}}{L_{t}\left(t\right)}\right),$$

where $\theta_{acc}>\theta \left(t\right)>\theta_{max}$ and $\theta_{max}$ is the maximum satellite elevation angle [23]. The θ(t) obtained in [23] is brought into Equation (6), and then the average channel capacity is calculated as

(7) $$\bar{C}\left(P_{i,j}\right)=\frac{1}{t_{\max}-t_{acc}}\int_{t_{acc}}^{t_{\max}}C(t,P_{i,j})dt$$

To satisfy the first condition, the average channel capacity $\bar{C}\left(P_{i,j}\right)$ of the communication link between user $u_{i}$ and satellite $S_{i,j}$ must satisfy $\bar{C}\left(P_{i,j}\right)\geq R_{v}$. We define $\bar{C_{Rv1}}$ as the minimum average channel capacity meeting this requirement, where $\bar{C_{Rv1}}=R_{v}$. However, the functional expression for $L_{t}\left(t\right)$ is so complex that it is not possible to write the original function of $C(t,P_{i,j})$, and only numerical methods (e.g., Simpson’s integral or trapezoidal methods) can be used to calculate the average channel capacity. This also makes it impossible to directly derive the required minimum power $P_{Rv1}$ with the $\bar{C_{Rv1}}$ known. In this paper, an indirect mathematical method is used to derive the required power. First, a reference power $P_{0}$ is preset and substituted into Equation (6). The value of $P_{0}$ can be arbitrarily selected from common power levels in LEO satellite systems, which does not affect subsequent algorithms. In simulations, we set $P_{0}$ = 25 dBW. By combining Equation (6) with Equation (7), the reference average channel capacity $\bar{C_{0}}$ corresponding to $P_{0}$ can be calculated:

(8) $$\bar{C_{0}}=\frac{1}{t_{\max}-t_{acc}}\int_{t_{acc}}^{t_{\max}}B\log_{2}\left(1+\frac{G_{tot}}{N}\cdot \frac{P_{0}}{L_{t}\left(t\right)}\right)dt$$

Using the trapezoidal rule for definite integrals, the definite integral in Equation (8) is rewritten in summation form:

(9) $$\bar{C_{0}}=\frac{1}{t_{\max}-t_{acc}}\sum_{k=1}^{K}B\log_{2}\left(1+\frac{G_{tot}}{N}\cdot \frac{P_{0}}{L_{t}\left(t_{acc}+\left(k-1\right)\Delta t\right)}\right)\Delta t$$

After moving the constant term outside the logarithm to the left-hand side, the right-hand side leverages the logarithmic identity by expressing the sum of multiple logarithms as the logarithm of the product of their arguments:

(10) $$\frac{\bar{C_{0}}\cdot \left(t_{\max}-t_{acc}\right)}{B\Delta t}=\log_{2}\left(\prod_{k=1}^{K}\left(1+\frac{G_{tot}}{N}\cdot \frac{P_{0}}{L_{t}\left(t_{acc}+\left(k-1\right)\Delta t\right)}\right)\right)$$

(11) $$2^{\frac{\bar{C_{0}}\cdot \left(t_{\max}-t_{acc}\right)}{B\Delta t}}=\prod_{k=1}^{K}\left(1+\frac{G_{tot}}{N}\cdot \frac{P_{0}}{L_{t}\left(t_{acc}+\left(k-1\right)\Delta t\right)}\right)$$

$P_{Rv1}$ and $\bar{C_{Rv1}}$ also satisfy the relationship in Equation (11), just as $P_{0}$ and $C_{0}$ do:

(12) $$2^{\frac{\bar{C_{Rv1}}\cdot \left(t_{\max}-t_{acc}\right)}{B\Delta t}}=\prod_{k=1}^{K}\left(1+\frac{G_{tot}}{N}\cdot \frac{P_{Rv1}}{L_{t}\left(t_{acc}+\left(k-1\right)\Delta t\right)}\right)$$

Let $\bar{C_{Rv1}}=d\cdot \bar{C_{0}}$, where *d* is the ratio of the minimum channel capacity $\bar{C_{Rv1}}$ to the reference channel capacity $\bar{C_{0}}$. We will later discuss the relationship between *d*, $P_{Rv1}$, and $P_{0}$, and we will estimate $P_{Rv1}$ using $P_{0}$ and *d*. Substituting $\bar{C_{Rv1}}=d\cdot \bar{C_{0}}$ into the left-hand side of Equation (12) , we obtain

(13) $$2^{\frac{\bar{C_{Rv1}}\cdot \left(t_{\max}-t_{acc}\right)}{B\Delta t}}=2^{\frac{d\cdot \bar{C_{0}}\cdot \left(t_{\max}-t_{acc}\right)}{B\Delta t}}={\left(2^{\frac{\bar{C_{0}}\cdot \left(t_{\max}-t_{acc}\right)}{B\Delta t}}\right)}^{d}$$

Substituting Equation (12) into the left-hand side of Equation (13), and substituting Equation (11) into the right-hand side of Equation (13), we obtain

(14) $$\prod_{k=1}^{K}\left(1+\frac{G_{tot}}{N}\cdot \frac{P_{Rv1}}{L_{t}\left(t_{acc}+\left(k-1\right)\Delta t\right)}\right)=\prod_{k=1}^{K}{\left(1+\frac{G_{tot}}{N}\cdot \frac{P_{0}}{L_{t}\left(t_{acc}+\left(k-1\right)\Delta t\right)}\right)}^{d}$$

In next-generation LEO satellite communication systems for broadband services, the value of $G_{tot}\cdot P_{0}/\left(L_{t}\left(t_{acc}+\left(k-1\right)\Delta t\right)\cdot N\right)$ in Equation (13) is positive and often approaches 0. Therefore, Equation (13) can be simplified using the following equivalent infinitesimal formula:

(15) $${\left(1+ax\right)}^{b}-1∼abx$$

where *a* and *b* are constants. According to the equivalent infinitesimal property, when *x* approaches 0 in Equation (15), we have ${\left(1+ax\right)}^{b}-1=abx$, i.e., ${\left(1+ax\right)}^{b}=1+abx$. Letting $ax=G_{tot}\cdot P_{0}/\left(L_{t}\left(t_{acc}+\left(k-1\right)\Delta t\right)\cdot N\right)$ and b=d, we obtain

(16) $${\left(1+\frac{G_{tot}}{N}\cdot \frac{P_{0}}{L_{t}\left(t_{acc}+\left(k-1\right)\Delta t\right)}\right)}^{d}\approx 1+d\cdot \frac{G_{tot}}{N}\cdot \frac{P_{0}}{L_{t}\left(t_{acc}+\left(k-1\right)\Delta t\right)}$$

Substituting Equation (16) into the right-hand side of Equation (14), we find that when $P_{Rv1}$ satisfies $d\cdot P_{0}=P_{Rv1}$, Equation (14) holds. Thus, we establish an indirect method to estimate $P_{Rv1}$ as follows:

(17) $$P_{R_{V}}\approx d\cdot P_{0}=\frac{\bar{C_{R_{V}}}}{\bar{C_{0}}}\cdot P_{0}$$

**Figure 4:**
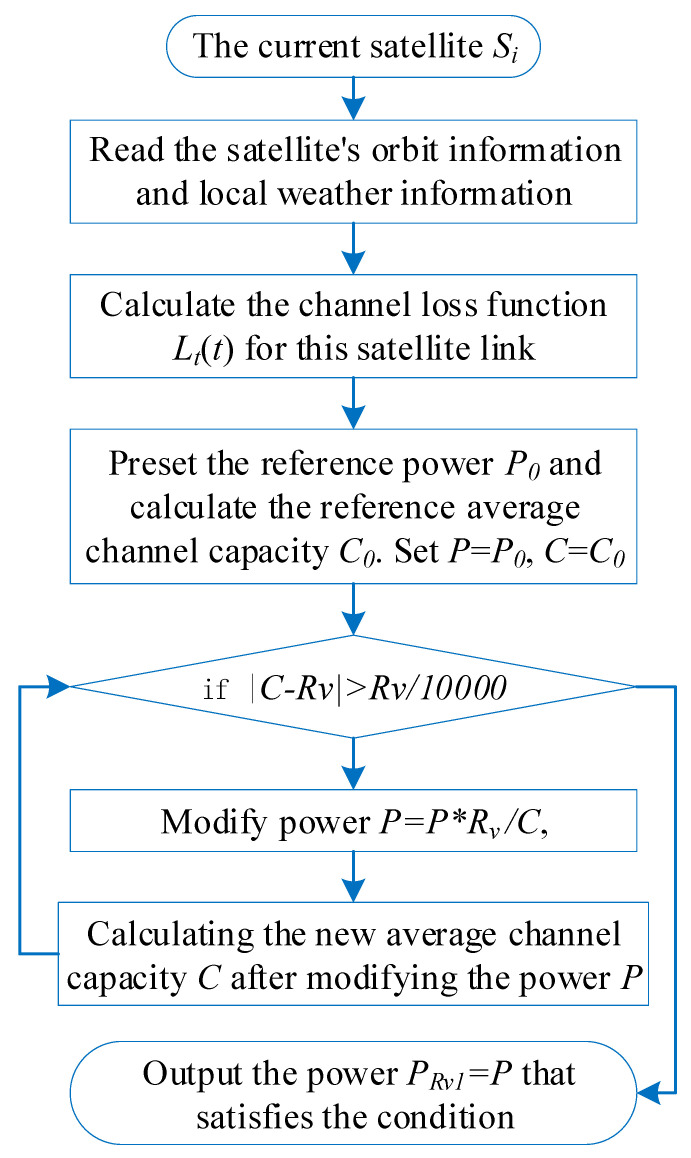
Structure of the algorithm for minimum power $P_{Rv1}$ satisfying the first condition.

The indirect calculation method in Equation (17) for estimating the minimum power $P_{Rv1}$ satisfying the first condition is an approximation. Although it provides $P_{Rv1}$ values close to the true minimum power that strictly satisfies $\bar{C_{Rv1}}=R_{v}$, a small discrepancy remains. In the implementation of the algorithm, the algorithm can be iterated several times to bring the calculated user access power, $P_{i,j}$, closer to the required minimum power. Specifically, after estimating $P_{Rv1}$ using the preset reference power $P_{0}$ through the aforementioned scheme, this estimated value is set as the updated reference power $P_{0}$ to recalculate the new reference average channel capacity $\bar{C_{0}}$. The updated $P_{Rv1}$ is then re-estimated using Equation (17). So far, we have used the above method to find the power threshold $P_{Rv1}$ to satisfy the first condition. In other words, the power of the user $u_{i}$, in conjunction with the satellites $S_{i,j}$, cannot be less than $P_{Rv1}$. The algorithm flow chart of $P_{Rv1}$ is shown in Figure 4.

**Figure 5:**
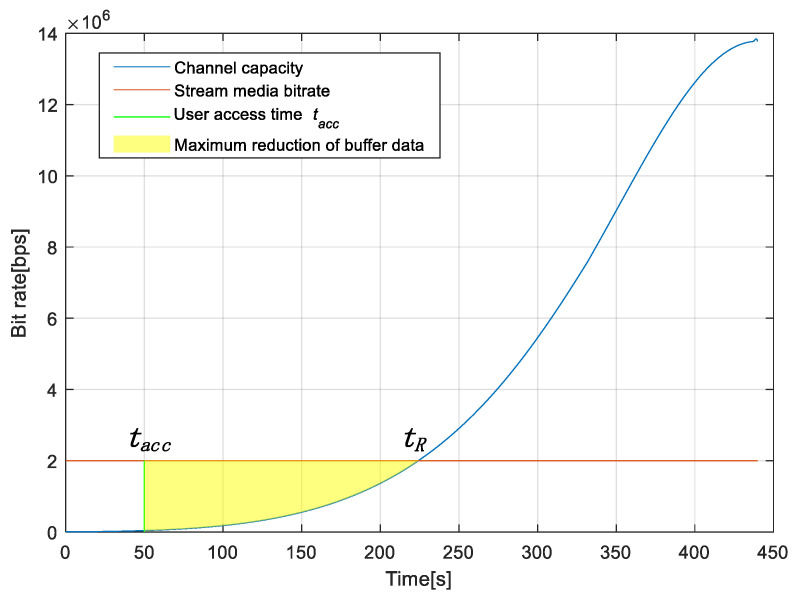
Graph of input and output rate functions for user buffers.

In order to satisfy the second condition, we need to analyze the changes in the streaming buffer on the user’s terminal. As shown in Figure 5, the channel capacity is the input rate of the streaming data in the buffer, and the playback bitrate of the stream is the output rate of the streaming data in the buffer. We assume that the user accesses the satellite at the time $t_{acc}$ and the channel capacity of the user is equal to the streaming bitrate $R_{v}$ at the time $t_{R}$.

Because the channel capacity increases as the satellite elevation angle increases, the channel capacity may be less than the streaming bitrate when the user terminal first accesses. So that, from $t_{acc}$ to $t_{R}$, the amount of data in the user’s buffer will decrease. When the satellite is operating at the time $t_{R}$, the reduction of data in the buffer will reach the maximum value, which is the integral value of the yellow area in Figure 5. The remaining data in the user’s buffer $H_{rem}$ must therefore exceed the maximum buffer data reduction to satisfy the second condition. It is expressed in the following mathematical formula:

(18) $$\int_{t_{acc}}^{t_{R}}C(t)dt>R_{v}\left(t_{R}-t_{acc}\right)-H_{rem}$$

A similar approach in the previous condition can be used to find the target power. In simple terms, first the power $P_{0}$ is preset, then the value of $t_{R0}$ is calculated, and then the preset power $P_{0}$ is brought into Equation (6) to compute equation $C_{0}\left(t\right)$ as a function of time t for the reference channel capacity. $C_{0}\left(t\right)$ is then taken into the left half of the inequality in Equation (18), and the total data volume in time $\left[t_{acc},t_{R0}\right]$ is calculated by numerical integration.

(19) $$\int_{t_{acc}}^{t_{R0}}C(t,P_{0})dt=R_{v}\left(t_{R0}-t_{acc}\right)-H_{rem}-\Delta H_{0}$$

Without loss of generality, we assume the preset power $P_{0}$ is sufficiently small, resulting in a reduction of $\Delta H_{0}$ in buffer data compared to $H_{rem}$ within the interval $\left[t_{acc},t_{R0}\right]$, as shown in Equation (19). This configuration fails to satisfy the condition in Equation (18). Consequently, we employ an estimation scheme analogous to the approach in Equation (17) to calculate the power $P_{1}=d\cdot P_{0}$ (d>1) that meets the second condition, as demonstrated in Equation (20):

(20) $$d=\frac{R_{v}\left(t_{R0}-t_{acc}\right)-H_{rem}}{\int_{t_{acc}}^{t_{R0}}C(t,P_{0})dt}$$

Substituting $P_{1}=d\cdot P_{0}$ and Equation (20) into the left-hand side of Equation (19), we derive the following process:

(21) $$\int_{t_{acc}}^{t_{R0}}C(t,P_{1})dt=d\cdot \int_{t_{acc}}^{t_{R0}}C(t,P_{0})dt=R_{v}\left(t_{R0}-t_{acc}\right)-H_{rem}$$

In Equation (21), the relation $C\left(t,P_{1}\right)=d\cdot C\left(t,P_{0}\right)$ in the first step is derived from Equation (17). While Equation (21) appears to satisfy the second condition (i.e., Equation (18)), the inequality $C\left(t_{R0},P_{1}\right)=d\cdot C\left(t_{0},P_{0}\right)>C\left(t_{1},P_{0}\right)=R_{v}$ implies that when power increases to $P_{1}$, $t_{R0}$ no longer represents the time when the user’s channel capacity $C(t_{R0},P_{1})$ equals the streaming bit rate $R_{v}$. Let $t_{R1}$ denote the new time satisfying $C\left(t_{R1},P_{1}\right)=R_{v}$, which differs from $t_{R0}$. Thus, substituting $t_{R1}$ into the left-hand side of Equation (21) invalidates the equality, indicating that $P_{1}$ is not the minimum power satisfying Equation (18). To apply the previous iterative approach, we must further analyze whether updating $P_{0}=P_{1}$ in Equation (19) brings $\Delta H_{0}$ closer to 0. Given that C(t,P) increases with time *t*, we assume $C\left(t_{R1},P_{1}\right)=R_{v}$ ($t_{acc}<t_{R1}<t_{R0}$) and substitute it into the left-hand side of the inequality in Equation (18):

(22) $$\int_{t_{acc}}^{t_{R1}}C(t,P_{1})dt=R_{v}\left(t_{R1}-t_{acc}\right)-H_{rem}-\Delta H_{1}$$

For $\Delta H_{0}$ and $\Delta H_{1}$:

(23) $$\begin{array}{c} \Delta H_{0}=\int_{t_{acc}}^{t_{R0}}C(t,P_{1})dt-\int_{t_{acc}}^{t_{R0}}C(t,P_{0})dt\\ =(d-1)\cdot \int_{t_{acc}}^{t_{R0}}C(t,P_{0})dt\\ >(d-1)\cdot \int_{t_{R1}}^{t_{R0}}C(t,P_{0})dt\\ =\int_{t_{R1}}^{t_{R0}}C(t,P_{1})dt-\int_{t_{R1}}^{t_{R0}}C(t,P_{0})dt\\ >\int_{t_{R1}}^{t_{R0}}C(t,P_{1})dt-R_{v}\left(t_{R0}-t_{R1}\right)\\ =\Delta H_{1} \end{array}$$

From Equation (23), we observe $\Delta H_{0}>\Delta H_{1}$, indicating that using Equation (20) to compute $P_{1}=d\cdot P_{0}$ brings the updated power $P_{1}$ closer to the true minimum power satisfying Equation (18). This scheme can be iterated several times to estimate the minimum power. Let $P_{Rv2}$ denote the minimum power fulfilling Equation (18).

**Figure 6:**
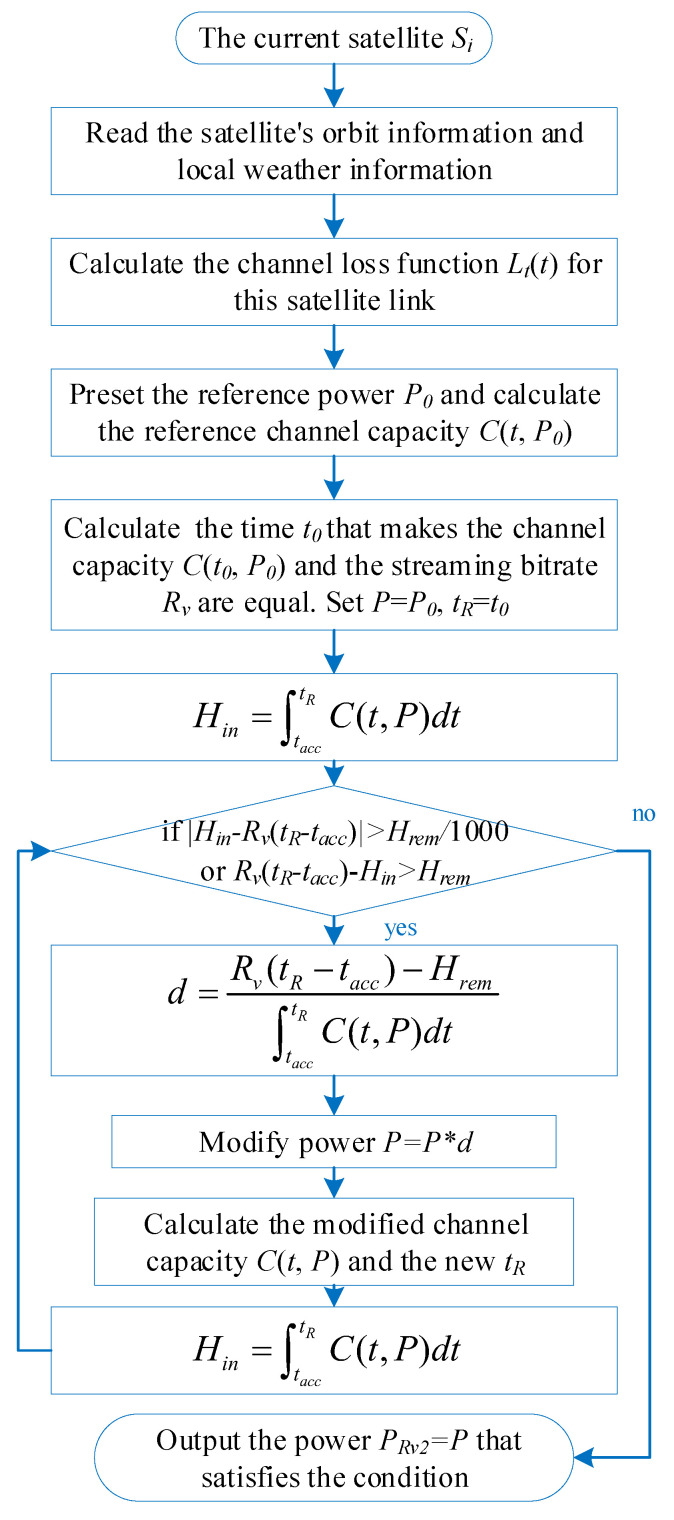
Structure of the algorithm for minimum power $P_{Rv2}$ satisfying the second condition.

Analogous to the iterative scheme for the first condition, we first preset a reference power $P_{0}$, compute $t_{R0}$, substitute it into Equation (20), and finally derive the adjusted power $P_{1}=d\cdot P_{0}$. By iteratively updating $P_{0}$ with $P_{1}$ and repeating the process, the computed power converges toward the target $P_{Rv2}$. The algorithm flowchart for $P_{Rv2}$ is shown in Figure 6.

Finally, the problem is formulated

(24) $$\begin{array}{c} CHO_{i,j}\left(P_{i,j},S_{i.j}\right)=-\alpha_{1}\gamma_{Pow}-\alpha_{2}\gamma_{Ch}\\ \left\{\begin{array}{c} \gamma_{Pow}=\frac{P_{i,j}+Poc_{i,j}}{P_{tot}},\gamma_{Pow}\leq 1\\ \gamma_{Ch}=\frac{1+Choc_{i,j}}{Ch_{tot}},\gamma_{Ch}\leq 1\\ P_{i,j}\geq \max\left(P_{Rv1},P_{Rv2}\right) \end{array}\right. \end{array},$$

where $CHO_{i,j}\left(P_{i,j},S_{i,j}\right)$ is the utility function when the user $u_{i}$ accesses the satellite $S_{i,j}$ with the power $P_{i,j}$ at the time of handover. $\gamma_{Pow}$ is the power loading loss function, which exceeds 1, meaning that the current satellite is underloaded and cannot provide the service; $Poc_{i,j}$ is the occupied power of the satellite, and $P_{tot}$ is the total power loading of the satellite. $\gamma_{Ch}$ is the channel loading loss function. $Choc_{i,j}$ is the number of occupied channels of the satellite, and $Ch_{tot}$ is the total number of available channels of the satellite. $\alpha_{1}$ and $\alpha_{2}$ are the respective weighting coefficients. The user $u_{i}$ traverses all the satellites of the set $S_{i}$ and then selects the satellite with the largest utility function among them for handover.

### 3.2. Access Scheme Design Based on Streaming Services

In the majority of cases, when the user’s service is in the initial buffering state, it means that it is in the buffering state at the beginning of the video playback. After initial buffering, the user terminal starts playing the streaming media. To ensure that the user’s service does not rebuffer after entering the playback state, power constraints similar to those in the previous handover section need to be met, i.e., an average channel capacity limit and a limit to prevent buffer exhaustion.

Typically, the initial buffering time of the video platform is very short. In order to avoid rebuffering soon after the end of buffering, it should be ensured that the channel capacity is not less than the user’s video bitrate $R_{v}$ at this time and the satellite is in the rising period after buffering is over. When the power of a user link satisfies this constraint, it also satisfies the power limit for the average channel capacity. Therefore, only the power limit after the initial loading is completed needs to be considered in the initial buffering state.

In order to calculate the minimum power $P_{Rv}$ meeting the above conditions, we need to use the approximate scheme of Equation (17). Similar to the handover environment, the user $u_{i}$ starts accessing at time $t_{acc}$ and the playback threshold of the initial buffering is known to be $T_{b}$ seconds, which means that the video client must load a video of the length of $T_{b}$ seconds before it can start playing. Thus, the power limit satisfies the following Equation (25):

(25) $$C(t_{acc}+T_{b},P)\geq Rv$$

Using the same approximation scheme as in Equation (17), we preset a reference power $P_{0}$ and substitute it into Equation (6) to calculate the reference channel capacity $C_{0}=C\left(t_{acc}+t_{b},P_{0}\right)$ after $t_{b}$ seconds. Through Equation (25), we determine the minimum required channel capacity $C_{Rv}=R_{v}$. These values are then substituted into Equation (17) to compute the minimum power $P_{Rv}$ satisfying the condition. Finally, analogous to the iterative estimation of $P_{Rv1}$ in Section 3.1, we perform several iterations to estimate $P_{Rv}$, bringing it closer to the true minimum power that strictly satisfies Equation (25).

Next, calculate the loss function $\gamma_{Deg}$ of the initial buffer. It is known that the current satellite residual power load is $P_{re}$, and when $P_{re}\geq P_{Rv}$, it means that the current satellite meets the minimum user demand. In other word, the limit of user link power is $P_{i,j}\in \left[P_{Rv},P_{re}\right]$. If $P_{re}<P_{Rv}$, this indicates that the current satellite cannot meet the user’s QoE demand, which is no rebuffering. To obtain the initial loading function $\gamma_{Deg}$, we refer to Equation (3) and rewrite it in a normalized way. When the user’s initial buffering time is less than 4.29 s, the user will have no QoE loss during the initial buffering phase. $\gamma_{Deg}$ can be derived at different powers $P_{i,j}\in \left[P_{Rv},P_{re}\right]$. If $P_{re}<P_{Rv}$ corresponding to different buffer times $t_{i,j}\in \left[t_{re},t_{Rv}\right]$.

After initial loading, we hope that the user can fill the buffer as much as possible to provide redundancy and resist channel instability. Therefore, in this paper, when the satellite is at maximum elevation, the data-filling amount of the user buffer is set as a reward function $\gamma_{Buff}$. Knowing that the maximum buffer capacity of the user terminal is $H_{buff}$, the minimum amount of data input required to just fill the buffer with streaming data when the satellite reaches the maximum elevation angle $\theta_{max}$ is $H_{sum}\left(P_{Buff}\right)$. It shown as Equation (26) below:

(26) $$H_{buff}+R_{v}\cdot \left(t_{\max}-t_{acc}\right)=H_{sum}\left(P_{Buff}\right)=\int_{t_{acc}}^{t_{\max}}C\left(t,P_{Buff}\right)$$

$t_{max}$ is the time at which the satellite reaches its maximum elevation angle, and tmax can be calculated [23]. $P_{Buff}$ can be calculated by the approximate scheme of Equation (17). We then set the handover threshold $H_{Thre}$ to the amount of streaming data in the buffer corresponding to a reward function of 0 at the time $t_{max}$. In other words, the reward function is 0 when the user power $P_{i,j}$ satisfies that the buffer at the user terminal is just filled to the handover threshold $H_{Thre}$ at the time $t_{max}$. It shown as Equation (27) below:

(27) $$H_{Thre}+R_{v}\cdot \left(t_{\max}-t_{acc}\right)=H_{sum}\left(P_{Thre}\right)=\int_{t_{acc}}^{t_{\max}}C\left(t,P_{Thre}\right)$$

$P_{Thre}$ can also be calculated by the approximate scheme of Equation (17). Thus, $\gamma_{Buff}$, the reward function of terminal buffer data volume when the satellite moves to the position of the maximum elevation angle, is

(28) $$\gamma_{Buff}=\min\left(\frac{P_{i,j}-P_{Thre}}{P_{Buff}-P_{Thre}},1\right)$$

$\gamma_{Buff}$ uses $P_{Buff}$ to normalize the user’s power $P_{i,j}$. As shown in Equation (28), the reward function value is 1 when $P_{i,j}\geq P_{Buff}$, which means that the user’s reward function does not increase with power when the power is too high. This is because the buffer will discard newly received streaming packets due to overflow. When $P_{i,j}<P_{Thre}$, the reward function value is negative. This means that the current power is too low, causing the channel capacity to be too low, so that when the satellite reaches its highest point, the amount of streaming data in the user’s buffer is still below the threshold that triggers the handover. Equation (29) is the problem formulation for the access scheme:

(29) $$\begin{array}{c} CAcc_{i,j}\left(P_{i,j},S_{i,j}\right)=\beta_{1}\gamma_{Buff}-\beta_{2}\gamma_{Pow}-\beta_{3}\gamma_{Ch}-\beta_{4}\gamma_{Deg}\\ \left\{\begin{array}{c} \begin{array}{cc} \gamma_{Pow}=\frac{P_{i,j}+Poc_{i,j}}{P_{tot}} & \gamma_{Pow}\leq 1 \end{array}\\ \begin{array}{cc} \gamma_{Ch}=\frac{1+Choc_{i,j}}{Ch_{tot}} & \gamma_{Ch}\leq 1 \end{array}\\ \begin{array}{c} \begin{array}{cc} \gamma_{Deg}=\frac{\max(\min\left(0.29\times \mathrm{lg}\left(t_{i,j}-3.29\right),4\right),0)}{4} & t_{i,j}>4.29 \end{array}\\ \gamma_{Buff}=\min\left(\frac{P_{i,j}-P_{Thre}}{P_{Buff}-P_{Thre}},1\right) \end{array}\\ P_{i,j}\in \left[P_{Rv},P_{re}\right] \end{array}\right. \end{array},$$

where $CAcc_{i,j}\left(P_{i,j},S_{i,j}\right)$ is the utility function when the user ui accesses the satellite $S_{i,j}$ with the power $P_{i,j}$ at the handover. $\gamma_{Pow}$ and $\gamma_{Ch}$ have the same meanings as in Equation (24). $\gamma_{Deg}$ is the loss function at the user’s initial buffering. $\gamma_{Buff}$ is the reward function of terminal buffer data volume when the satellite reaches the position of the maximum elevation angle; $P_{buff}$ is the power required for the data to exactly fill the buffer, and $P_{Thre}$ is the power required to buffer data up to $H_{Thre}$, which is the threshold value of the remaining data in the buffer that triggers handover when the satellite descends. $\beta_{1}$, $\beta_{2}$, $\beta_{3}$ and $\beta_{4}$ are the respective weighting coefficients. In this paper, we set $\beta_{1}=\beta_{2}=\beta_{3}=\beta_{4}=1$. The user $u_{i}$ traverses all the satellites of the set $S_{i}$ and then selects the satellite with the largest utility function among them to access.

### 3.3. Power Occupation Handover Scheme Design Based on Streaming Services

When satellite handover fails multiple times and persistent streaming service consumption causes the buffer data to fall below the power occupation threshold $H_{oc}$, the system enters the power occupation handover module. This module allocates a portion of another user’s power to the current user in this module to meet the minimum power requirement of this user. On one hand, when the satellite moves from a low-elevation position to a high-elevation position, channel loss decreases substantially, leading to significantly increased channel capacity that far exceeds current streaming bitrate requirements. On the other hand, users with abundant buffer data can avoid rebuffering by ceding partial power to other users whose buffers are nearly depleted. For instance, when user $u_{1}$ exhausts its buffer due to repeated handover failures and enters rebuffering, the system allocates a portion of power from another user $u_{2}$’s link to $u_{1}$, preventing playback interruption. After obtaining sufficient power, $u_{1}$ can continue replenishing its buffer data, while $u_{2}$ may experience buffer depletion due to the power cession. In essence, this allows user $u_{2}$ to bear the negative impact of high satellite network load on behalf of $u_{1}$. However, as revealed by the MOS scoring mechanism in Section 3, the user QoE remains unaffected as long as playback continues without rebuffering, even if the current channel capacity fails to meet service demands. Therefore, the QoE of $u_{2}$ will not degrade in the short term as long as its buffer is not exhausted.

**Figure 7:**
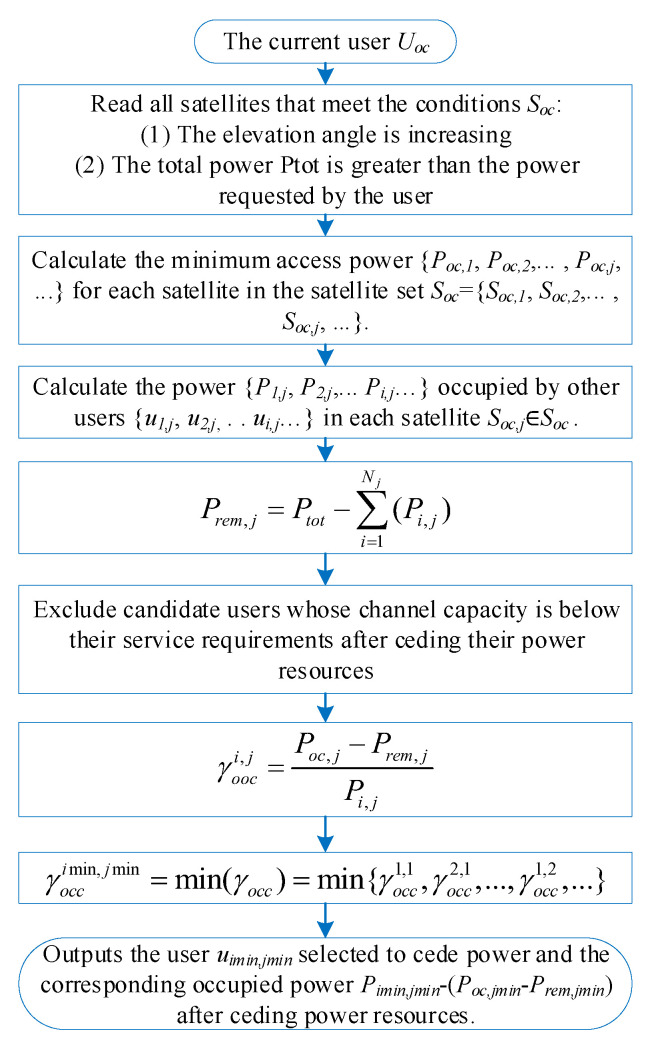
Structure of the algorithm for power occupation handover.

When the user $U_{oc}$ repeatedly fails to handover and enters the power occupation module, the user $U_{oc}$ first uses all satellites $S_{oc}=\left\{S_{oc,1},S_{oc,2},...,S_{oc,j},...\right\}$ in the rising stage as candidates . Each candidate satellite $S_{oc,j}$ is used by other users $\left\{u_{1,j},u_{2,j},...,u_{i,j},...\right\}$ occupying the power resources $\left\{P_{1,j},P_{2,j},...,P_{i,j},...\right\}$. Eliminate the users in $\left\{u_{1,j},u_{2,j},...,u_{i,j},...\right\}$ with channel capacity below the streaming bitrate. The minimum power required for user $U_{oc}$ to access each candidate satellite $S_{oc,j}$ is calculated, and this minimum power $\left\{P_{oc,1},P_{oc,2},...,P_{oc,j},...\right\}$ calculation scheme is referred to the handover scheme in Section 3.1. In our scheme, only one satellite cedes power resources. Calculate the proportion $\gamma_{ooc}$ of power being used by each user served by each candidate satellite that needs to be ceded by each of them. The user link with the smallest proportion of power is selected to yield power to the user $U_{oc}$ who is currently in the power occupation handover state.

(30) $$\gamma_{ooc}^{i,j}=\frac{P_{oc,j}-P_{rem,j}}{P_{i,j}}$$

(31) $$P_{rem,j}=P_{tot}-\sum_{i=1}^{N_{j}}\left(P_{i,j}\right)$$

where $\gamma_{occ}^{i,j}\in \gamma_{occ}=\left\{\gamma_{occ}^{1,1},\gamma_{occ}^{2,1},...,\gamma_{occ}^{1,2},...\right\}$ denotes the ratio of power ceded by user $u_{i,j}$ (served by satellite $S_{oc,j}$) to $U_{oc}$, which is defined as the ceded power divided by the user’s own consumed power $P_{i,j}$. The user link with the smallest $\gamma_{occ}$ is selected for power transfer. $P_{oc,j}$ denotes the power required for $U_{oc}$ to access $S_{oc,j}$, while $P_{rem,j}$ (Equation (31)) is the residual power of $S_{oc,j}$, which is calculated by subtracting the total occupied power from the satellite’s total power $P_{tot}$. Users whose channel capacity falls below service requirements after power cession are excluded. Finally, the user $u_{i,j}$ with the smallest $\gamma_{occ}$ cedes power to $U_{oc}$, which connects to the selected satellite $S_{oc,j}$. The power occupation handover algorithm flowchart is shown in Figure 7.

## 4. Performance Evaluation

In this section, we will use the simulation system built in this paper to perform simulations and evaluate the performance of the proposed QoE access and handover method for subscriber streaming services through simulations.

### 4.1. Simulation Parameters

We simulate the OneWeb constellation in the simulation software to generate the ephemeris data required by our proposed algorithm. The altitude of this LEO satellite is 1200 km, and the rest of the parameters are shown in Table 3. The weather parameters are predefined into five levels, which are ranked by their degradation impact on the communication channel from severe (Level 5) to optimal (Level 1). Specifically, Level 5 is rainy conditions (most severe), and Level 1 is clear sky (optimal conditions). Each user is assigned a unique weather array containing indices [1,5] that define their weather level variation sequence. Whenever a user attempts to access a new satellite, the system advances to the next index in their weather array, simulating time-varying weather conditions. Detailed definitions of all five weather levels are provided in the Appendix A. The simulation time slot is set to 1 s, and the simulation time length is set to 1 h. Considering the randomness of the user when using the streaming service, we divide the user’s video playback time into several segments with several minutes between each segment. Our simulations initially revealed that under low-load conditions, both our proposed method and baseline approaches achieved near-maximum MOS scores, making performance comparisons indistinguishable. To create meaningful evaluation conditions, we strategically configure a high-load environment by reducing the satellite downlink power to 24 dBW.

For performance comparison, there are few schemes to analyze the QoE streaming media service in an LEO communication system. Therefore, two traditional handover schemes, i.e., balanced power and channel load, and the longest visible time are selected for comparison.

### 4.2. Simulation Results

**Figure 8:**
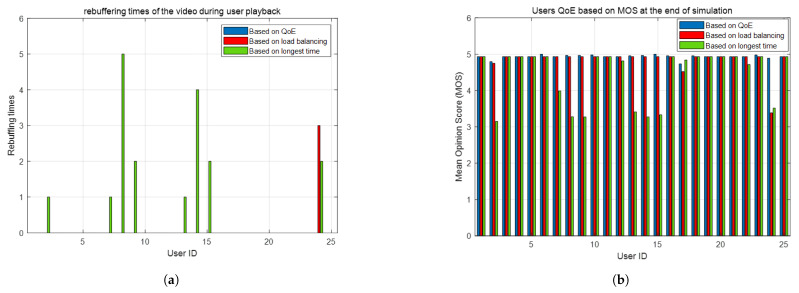
These are two figures from two simulations: (**a**) number of rebuffers during user playback; (**b**) users’ QoE based on MOS.

The performance of the streaming service in the LEO satellite network is shown in Figure 8a,b. Figure 8a shows the total amount of rebuffering per user at the end of the simulation. From the analysis in Section 3, it can be seen that rebuffering can have a very negative impact on the user’s QoE, so in environments with high load pressure, fewer buffers mean a better user experience. As can be seen from Figure 8a, the QoE-based scheme proposed in this paper does not have a rebuffering event; the longest time scheme and load-balancing scheme both have more rebuffering times than the QoE-based scheme.

Figure 8b displays the MOS score for each user at the end of the simulation, which is illustrated in Section 3 to be reflective of the user’s QoE. As can be seen from Figure 8b, for only user number 17, the MOS for the QoE-based scheme is lower than the MOS for the longest time-based scheme, whereas for all other users, the QoE-based scheme has a higher MOS than the other two schemes. This means that the QoE-based scheme will perform better than the load-balancing-based scheme and the longest visible time-based scheme in terms of the QoE affected by the initial buffering and rebuffing of the user.

From the above three sets of performance comparisons, it can be seen that the QoE-based scheme for streaming media services proposed in this paper performs better than the load-balancing-based scheme in traditional satellite communication. Not only does the QoE-based scheme outperform the other two traditional schemes in the playback stalling event, which affects the user experience the most, the QoE-based scheme also outperforms the other two traditional schemes on the MOS, which measures the overall QoE. This is because the handover and access scheme in traditional satellite communications does not take into account the characteristics of the user’s service. For example, the streaming service has a buffer, which is the ability to resist channel instability and network congestion. Using the cache in the buffer to appropriately reduce the required power can reduce the load pressure in the satellite communication environment.

## 5. Conclusions

In this paper, the QoE for streaming media services is investigated in a future system for broadband LEO satellites. A simulation system for streaming media transmission and playback in a satellite communication system is constructed to evaluate the performance of the handover and access scheme proposed in this paper for the QoE of streaming media services. There are few comprehensive simulations of the downlink transmission process of streaming media services under satellite communication systems. Therefore, the first objective of this study is to build a simulation system in a multi-satellite system where multiple users can transmit streaming media services and simulate the playback state in order to realize the functions including initial buffering, re-buffering, buffer, etc., and record the amount of initial buffering and time as well as the number of instances of re-buffering. This simulation system is very useful for the verification and evaluation of more environments for streaming media services in satellite environments in the future. In addition to this, the second objective of this research is to design the QoE-based access and handover scheme for streaming services and evaluate its performance using the built-in simulation system. Taking advantage of the redundant nature of the streaming buffer, using the cache in the buffer to appropriately reduce the power required can reduce load stress in a satellite communication environment. For users whose streaming media playback is about to stall due to the constraints of LEO network resources, the power occupation handover module utilizes the idea of trading time for communication resources to allow other users’ buffers to be consumed in place of the current user’s buffer so that both the current user and the substituted user will not be stalled. From the simulation results, the access and handover scheme proposed in this paper can better adapt to satellite communication environments by designing for the buffer and high load characteristics of streaming media, occupying fewer resources and resisting to some extent the congestion caused by too many satellite users. This can provide a lot of help for the popularization of multimedia services in satellite environments for mobile 6G in the future.

From the existing work and simulations, we find that accessing the satellite at low elevation angles tends to require much more power than at high elevation angles. This leads to the fact that after access to a satellite, when the satellite reaches a high elevation angle position, the channel capacity of the star–ground link increases much more than at the time of access. This often leads to serious buffer overflow for user streaming services, which is a very serious waste. Therefore, our next step is to consider how to allocate power resources. Adjusting power dynamically after the user has accessed the satellite, rather than just allocating power at the time of access, requires a combination of considering the timing of the power adjustment and how to use the buffer’s data as well as the buffer’s own resistance to sudden fading.

## Figures and Tables

**Table 1:** Link loss factors and their symbols.

Symbol	Loss Factors	Symbol	Loss Factors
$L_{f}$	free space loss	$A_{c}$	cloud attenuation
$A_{o}$	oxygen attenuation	$A_{r}$	rainfall attenuation
$A_{w}$	water vapor attenuation	$A_{s}$	tropospheric scintillation

**Table 2:** Values of the coefficients of Equations (3) and (4).

$s_{1}$	$s_{2}$	$s_{3}$	$s_{4}$	$d_{1}$	$d_{2}$
−1.72	−0.04	−0.36	1.66	0.29	−3.29

**Table 3:** Some parameters of the simulation system.

Parameter	Value
Number of users	25
Satellite downlink transmit power $P_{tot}$	24 dBW
Satellite operating frequency band *f*	30 GHz
Minimum elevation angle for satellite service	$10^{{}^{\circ}}$
Number of channels available for satellite downlink *N*	10
Transmit antenna gain	29 dB
Receiving antenna gain	5 dB
Bandwidth *B*	500 MHz
Subscriber service bitrate $R_{v}$	6 Mbps·1 + 4 Mbps·8 + 2 Mbps·13 + 1 Mbps·3
Buffer capacity limit $H_{buff}$	60 s· $R_{v}$
Buffer handover threshold $H_{Thre}$	30 s· $R_{v}$
Playback threshold time $T_{b}$ of the initial buffering	5 s
Power occupation threshold $H_{oc}$	10 s· $R_{v}$

## Data Availability

Data are contained within the article.

## Appendix A

The following are detailed parameters of different levels of weather represented by numbers 5 to 1.

**Table A1:** Weather parameters for levels 1–5.

Parameters	5	4	3	2	1
Relative humidity	0.95	0.86	0.62	0.5	0.3
Saturated vapor pressure (hPa)	42.4	42.4	33.6	42.4	40
Saturated vapor density (g/m^3^)	30.3	30.3	24.3	31	28.5
Point rainfall rate (mm/h)	10	0	0	0	0
Total columnar content of the cloud liquid water (kg/m^3^)	5	4	2	0.5	0
Atmospheric pressure (hPa)	1000	1006	1010	1000	946
Temperature (°C)	30	30	26	30	29
